# Developing Instructional Support for MEDSReM-2 Through Human Factors Design Principles

**DOI:** 10.1093/geroni/igab046.883

**Published:** 2021-12-17

**Authors:** Tracy Mitzner, Maurita Harris, Kenneth Blocker, Mimi Trinh

**Affiliations:** 1 Georgia Institute of technology, Atlanta, Georgia, United States; 2 University of Illinois at Urbana Champaign, Champaign, Illinois, United States; 3 University of Illinois at Urbana-Champaign, Champaign, Illinois, United States; 4 University of Illinois Urbana-Champaign, Champaign, Illinois, United States

## Abstract

Appropriate instruction is critical for ensuring the MEDSReM-2 system (i.e., smartphone app, blood pressure monitor, online portal) will be easily and effectively used and will, therefore, be more likely to be adopted. We will present our iterative processes for developing instructional support for MEDSReM 2 using human factors design principles (e.g., task analyses, comparative analyses, expert evaluation of mock-ups with screen flows). The instructional supports include user manuals, videos, as well as instructions within the MEDSReM 2 app. We will also highlight design principles used to empower the user and the benefits of using an interdisciplinary approach (i.e., gerontology, cognitive psychology, educational psychology, design, community health) to develop instructional support for older adult users.

